# miRNA–mRNA–protein dysregulated network in COPD in women

**DOI:** 10.3389/fgene.2022.1010048

**Published:** 2022-11-17

**Authors:** Chuan Xing Li, Jing Gao, C. Magnus Sköld, Åsa M. Wheelock

**Affiliations:** ^1^ Respiratory Medicine Unit, Department of Medicine, Centre for Molecular Medicine, Karolinska Institutet, Stockholm, Sweden; ^2^ Heart and Lung Centre, Department of Pulmonary Medicine, University of Helsinki and Helsinki University Hospital, Helsinki, Finland; ^3^ Department of Respiratory Medicine and Allergy, Karolinska University Hospital, Stockholm, Sweden

**Keywords:** chronic obstructive pulmonary disease, multi-omics integration, miRNA, miRNA dysregulation, miRNA–mRNA–protein network

## Abstract

**Rationale:** Chronic obstructive pulmonary disease (COPD) is a complex disease caused by a multitude of underlying mechanisms, and molecular mechanistic modeling of COPD, especially at a multi-molecular level, is needed to facilitate the development of molecular diagnostic and prognostic tools and efficacious treatments.

**Objectives:** To investigate the miRNA–mRNA–protein dysregulated network to facilitate prediction of biomarkers and disease subnetwork in COPD in women.

**Measurements and Results:** Three omics data blocks (mRNA, miRNA, and protein) collected from BAL cells from female current-smoker COPD patients, smokers with normal lung function, and healthy never-smokers were integrated with miRNA–mRNA–protein regulatory networks to construct a COPD-specific dysregulated network. Furthermore, downstream network topology, literature annotation, and functional enrichment analysis identified both known and novel disease-related biomarkers and pathways. Both abnormal regulations in miRNA-induced mRNA transcription and protein translation repression play roles in COPD. Finally, the let-7-AIFM1-FKBP1A pathway is highlighted in COPD pathology.

**Conclusion:** For the first time, a comprehensive miRNA–mRNA–protein dysregulated network of primary immune cells from the lung related to COPD in females was constructed to elucidate specific biomarkers and disease pathways. The multi-omics network provides a new molecular insight from a multi-molecular aspect and highlights dysregulated interactions. The highlighted let-7-AIFM1-FKBP1A pathway also indicates new hypotheses of COPD pathology.

## 1 Introduction

Chronic obstructive pulmonary disease (COPD) is a complex disease representing an umbrella diagnosis caused by a multitude of underlying mechanisms, including environmental exposures, genetic predispositions, and developmental factors (Merikallio et al., 2020; Nouws et al., 2021). Molecular mechanistic modeling of COPD, especially at a multi-omics level, will therefore be essential in order to develop relevant diagnostic and treatment options for this constantly growing patient group (Li et al., 2018). miRNAs and their dysregulations in mRNA and protein expression have been proved to play important roles in the progression of COPD and other complex diseases (Szymczak et al., 2016; Canas et al., 2020). The availability of the miRNA–mRNA regulatory network and multi-omics expression profile at miRNA, mRNA, and protein levels, as well as newly developed computational approaches, provides an opportunity to systematically investigate the miRNA–mRNA–mRNA dysregulated network in COPD.

miRNAs and their dysregulations in mRNA and protein expression have been proved to play important roles in the progression of COPD and other complex diseases (Van Pottelberge et al., 2011; Ezzie et al., 2012; Liu et al., 2016; Qian et al., 2018; Canas et al., 2020). In particular, differentially expressed miRNA, mRNA, and their dysregulated interactions have been studied. Liu et al. identified potential COPD genes in the methylation–microRNA–MRNA–GO network. Qian et al. investigated miRNA–mRNA–lncRNA networks in non-smoking and smoking patients with COPD (Qian et al., 2018). Multi-omics integration and computational systems medicine approaches have been developed and applied in the subgrouping and biomarker identification of complex, heterogeneous diseases (Sathyanarayanan et al., 2020; Cheng et al., 2021; Li et al., 2022). Methods for differential co-expression networks to identify changes in disease or response to external perturbation are emerging in which the focus is on dysregulated network edges (regulations) instead of dysregulated nodes to assemble disease-related signatures (Xu et al., 2011b; van Dam et al., 2018; Savino et al., 2020). Specifically, we have shown that multi-omics integration analysis improves the power to define subgroups with a small sample size in COPD, which also indicates its potential ability to capture molecular modeling of disease (Li et al., 2018). miRNA and its dysregulation in COPD progression have been proved in multiple studies, and many miRNAs (clusters) have been listed (Canas et al., 2020). In our previous works, we have performed a series of studies ranging from the construction of a miRNA–miRNA synergistic network to prioritizing disease miRNA and miRNA–mRNA dysregulated bi-modules by network-based integration of miRNA and mRNA expression with a three-level hypergraph, namely, miRNA–miRNA synergistic network, miRNA–mRNA regulatory network, and functional gene network (Xu et al., 2011a; Xu et al., 2011b; Xu et al., 2013). Here, we defined the union of differential co-expression regulations from miRNA to target mRNA, mRNA to protein, and miRNA to protein, as a dysregulated network. We focused on exploring the regulatory changes from miRNAs to mRNAs and proteins with the integration of triple omics data, as well as the miRNA to mRNA targeting network. In this study, we extended this systematic method to identify a COPD-related miRNA–mRNA–protein dysregulated network by integration of the miRNA–mRNA–protein regulatory network with triple omics from the Karolinska COSMIC cohort of COPD.

## 2 Materials and methods

### 2.1 Clinical cohort

Omics data blocks from the Karolinska COSMIC cohort (ClinicalTrials.gov ID: NCT02627872), a three-group cross-sectional study (Kohler et al., 2013) (Li et al., 2018) with age- (45–65 years) and sex-matched groups of healthy never-smokers (“healthy”), smokers with normal lung function (“smokers”), and COPD patients (“COPD”; GOLD stage I–II/A–B; FEV1 = 51–97%; FEV1/FVC<70%), were utilized (see clinical parameters in Supplementary Table S1). The COPD group of the full cohort contained both current smokers and ex-smokers. For this study, only current-smoker COPD patients were included to limit confounding effects of acute smoking. Bronchoalveolar lavage (BAL) was collected as previously described (Kohler et al., 2013; Forsslund et al., 2014). Participants had no history of allergy or asthma, did not use inhaled or oral corticosteroids, and had no exacerbations for at least 3 months prior to study inclusion. Current smokers were matched in terms of smoking history (>10 pack-years) and current smoking habits (>10 cigarettes/day in the past 6 months). Current smoking status and abstinence for >8 h prior to BAL were verified through exhaled carbon monoxide (Sandberg et al., 2011). The study was approved by the Stockholm Regional Ethical Board (Case No. 2006/959-31/1), and participants provided their informed written consent.

### 2.2 miRNA, mRNA, and protein omics data blocks from BAL cells

Three omics data blocks from the same 20 female subjects (four healthy, 11 smokers, and five COPD) were utilized. RNA from BAL cells was isolated into two fractions containing small RNAs (including miRNAs) and large RNAs (containing mRNA) using the NucleoSpin® miRNA kit according to the manufacturer’s instructions (Macherey-Nagel, Düren, Germany) (Levanen, 2012; Balgoma et al., 2016). mRNA from BAL cells were hybridized to Agilent human whole-genome 4 × 44K ink-jet arrays containing a total of 41,000 probes corresponding to 19,596 Entrez genes. Small RNA was labeled with Cy3-CTP using the miRCURY LNA microRNA Power Labeling Kit (Exiqon Inc., Woburn, MA) and then hybridized to one-color Agilent custom UCSF multi-species 8 × 15 K ink-jet arrays (Agilent Technologies, miRNA, v3.6) containing 894 miRNAs. For both mRNA and miRNA microarrays, raw signal intensities were extracted using Feature Extraction v10.1 software (Agilent Technologies); no background subtraction was performed; and the median feature pixel intensity was used as the raw signal before normalization (Levanen, 2012; Levanen et al., 2013; Balgoma et al., 2016). Shotgun proteomics data from BAL cells were collected using isobaric tags for relative and absolute quantitation (iTRAQ) mass spectrometry (MS) (Yang et al., 2018a; Yang et al., 2018b). Peak integration of iTRAQ MS/MS spectra was performed using Proteome Discoverer 2.1 (Thermo Fisher Scientific) and searched against the UniProt human database (2015_12). Ratio data of samples to reference were log2-transformed. All data were log2-transformed and quantile-normalized in the R package limma in Bioconductor (Ritchie et al., 2015). All mRNA, miRNA, and protein identifiers were updated in the Ensembl BioMart database (2018–10) (Cunningham et al., 2022). The data collection platform and processing are the same as our previous work (Li et al., 2018).

### 2.3 Construction of the miRNA-mRNA-protein dysregulated network

**FIGURE 1:**
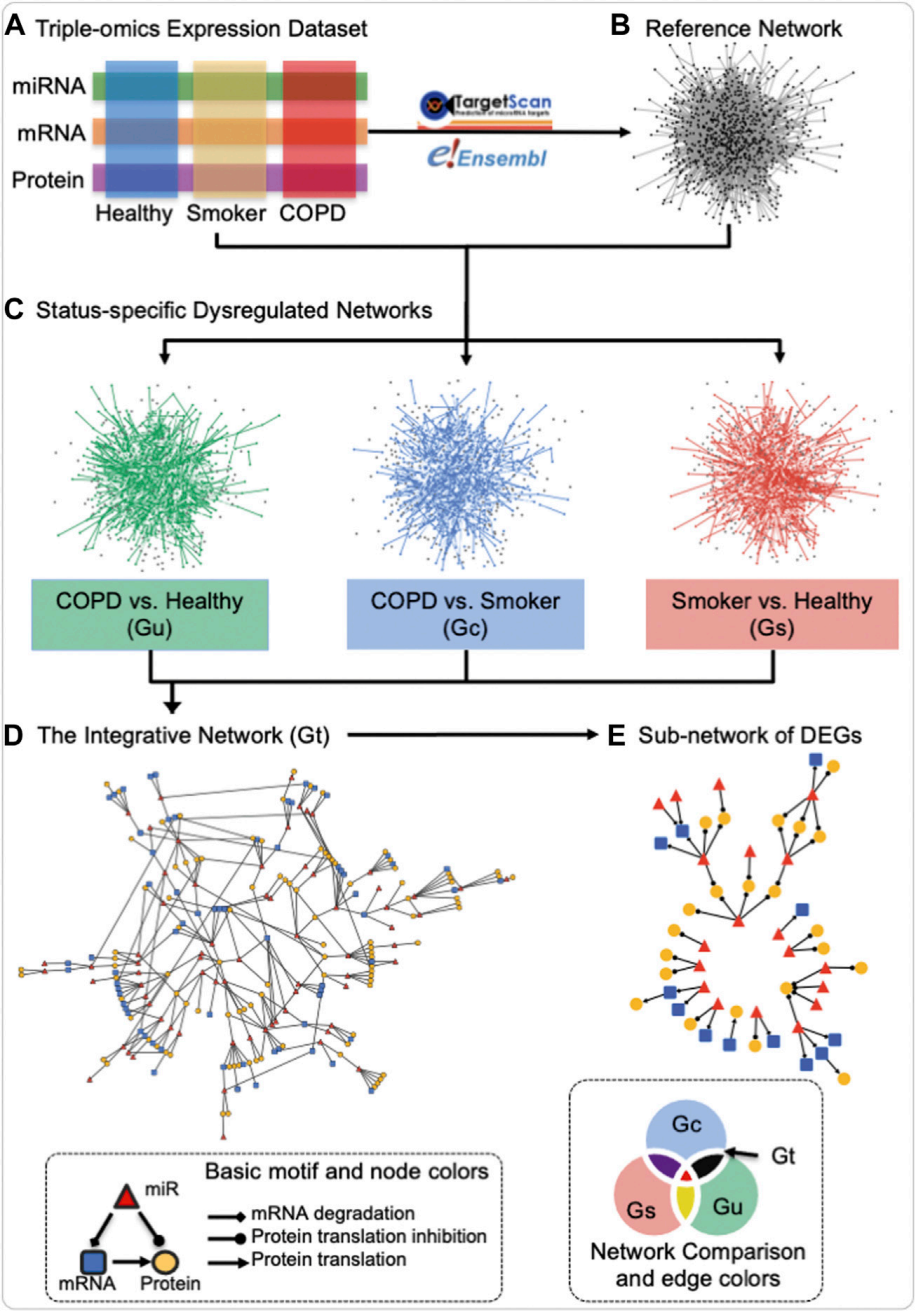
Schematic of the construction of the miRNA–mRNA–protein dysregulation network. Three data modalities (miRNA, mRNA, and protein) from the three groups of healthy never-smokers (healthy), current-smokers with mild-to-moderate COPD (COPD), and smokers with normal lung function (smokers) **(A)** were utilized for the construction of a reference network by mapping miRNA to mRNA regulation and mRNA to protein translation in TargetScan and Ensembl databases **(B)**, resulting in the union of three-node basic motifs (bottom left inset). Status-specific dysregulated networks from each contrast of interest were then extracted from differentially co-expressed interactions in each status comparison **(C)** An integrative dysregulated network **(D)** was then constructed using the network set operation illustrated in “Network Comparison” (bottom right inset), where Gt (black) represents the main contrast of interest for this investigation, namely, the difference between the “status-specific dysregulated networks” in “COPD vs. healthy” (Gu) and “smoker vs. healthy” (Gs), when intersected with the network of “COPD vs. smokers” (Gc). Finally, sub-networks containing differentially expressed genes (DEGs) were extracted for further investigation **(E)** In “Basic motif and node colors,” the node and edge shapes applied to all panels and the node colors used that of panel d and **(E)** In “Network Comparison and edge colors,” the colors for different networks corresponded to both edge and node in panel **(C)** The edge and node colors are grey and black in Reference Network of panel **(B)** Created using igraph in R and Cytoscape.

The miRNA–mRNA–protein dysregulated network is defined as a network with three types of nodes: 1) miRNA, 2) mRNA, and 3) protein and three types of directed edges: 1) from miRNA to mRNA as targeted regulation, 2) from mRNA to protein as translation, and 3) from miRNA to protein by bridging mRNA. The definition of a dysregulated network in this study is significantly differentially co-expressed patterns of two connected nodes between two statuses. As illustrated in the schematic of Figure 1, the construction of the miRNA–mRNA–protein dysregulated network includes four steps: first, three data modalities (miRNA, mRNA, and protein) from the three groups of “healthy”, “COPD”, and “smokers” (Figure 1A) were utilized to construct the reference network. The reference network includes three types of nodes (miRNA, mRNA, and protein) and three types of directed edges (miRNA regulation of mRNA based on the TargetScan database version 7.1 (Agarwal et al., 2015), mRNA translation to protein based on the Ensembl database version 84 (Howe et al., 2021), and miRNA potential regulation of protein inferred from the protein’s corresponding mRNA, based on transfer regulation from miRNA to mRNA to protein from TargetScan). It is the union of three-node basic motifs (Figure 1, bottom left inset). Second, “status-specific dysregulated networks” from each contrast of interest were then extracted from differentially co-expressed interactions in each status comparison (Figure 1C). For every two-status comparison, such as “COPD vs. healthy”, the edge weight is the absolute difference between the Pearson correlation coefficients of two connected nodes in each status, in accordance with the definition used in our previously published study on prostate cancer (Xu et al., 2011b). Subsequently, the corresponding false discovery rate (FDR) is estimated based on 10,000 permutations of the sample status (Storey and Tibshirani, 2003). The largest connected part in the reference network after filtering of edges based on FDR thresholds is defined as the status-specific dysregulated network. Third, the “integrative dysregulated network” (Figure 1D) was then constructed using the network set operation (illustrated in Network Comparison of Figure 1, bottom right inset). The integrative dysregulation network was constructed based on the difference between the “status-specific dysregulated networks” in “COPD vs. healthy” and “smoker vs. healthy” and then intersected with the network of “COPD vs. smokers.” Finally, the “sub-network of differentially expressed genes (DEGs)” was extracted from the integrative dysregulated network if both connected nodes were differentially expressed in either “COPD vs. smokers” or “COPD vs. healthy” (Figure 1E). DEGs were tested by the t-test to check if the variable’s expression fits normal distribution and homogenous variance among all subjects; otherwise, the Kruskal–Wallis rank-sum test was performed (*p*-value ≤ 0.05). Overall, the input of construction of a miRNA–mRNA–protein dysregulated network is the three omics data, links of miRNA to mRNA regulation and mRNA to protein mapping. It outputs three status-specific dysregulated networks, an integrative dysregulated network, and a sub-network of DEGs.

### 2.4 Topological analysis and motif identification

**FIGURE 2:**
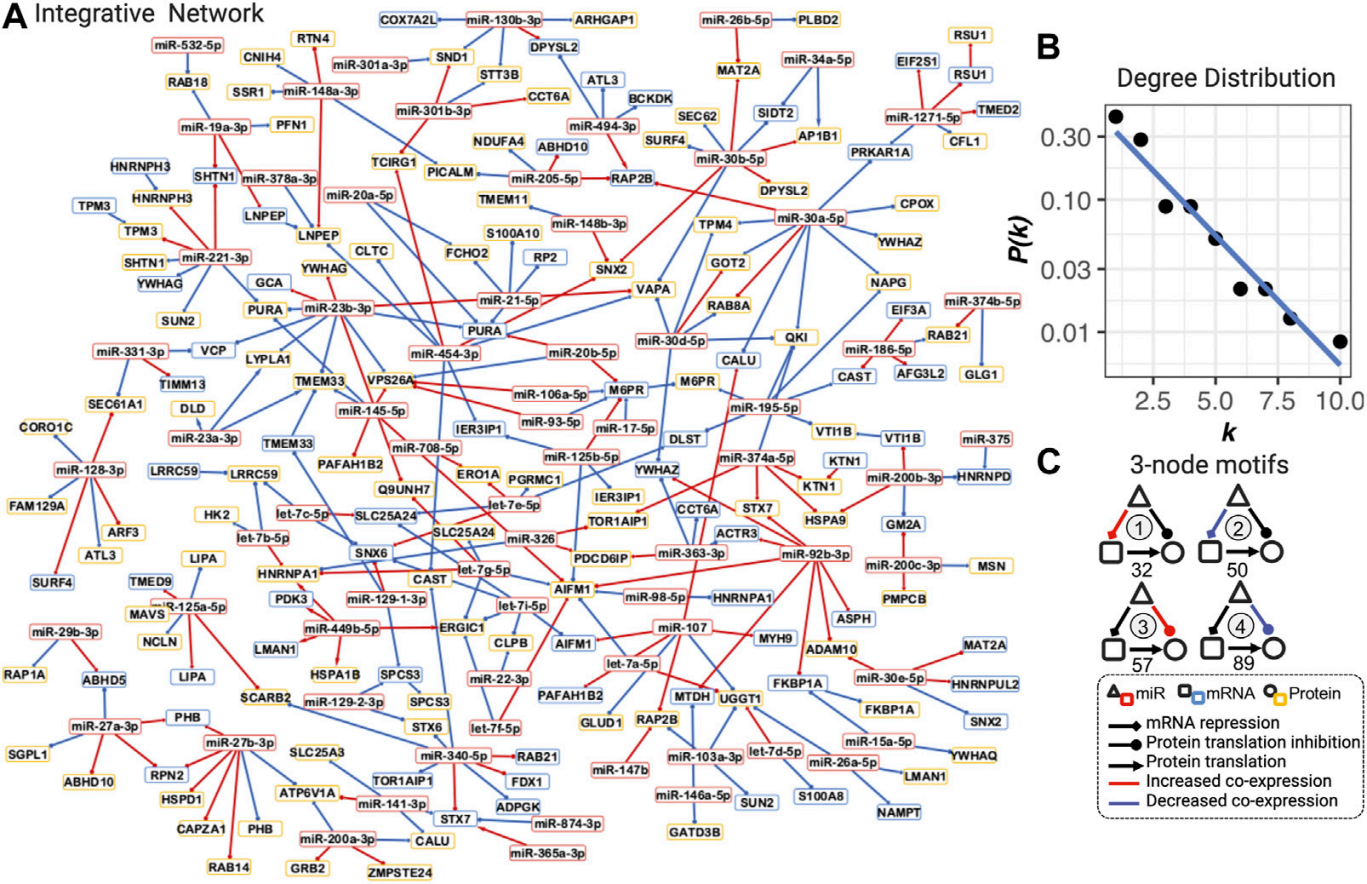
Integrative dysregulated network **(A)**, its degree distribution **(B)**, and three-node motifs and their counts **(C)**. **(A)** Integrative dysregulated network is a directed network from miRNA to mRNA, mRNA to protein, or from miRNA to protein (see legend inset, bottom right). Nodes with red, blue, and yellow borders represent miRNA, mRNA, and protein, respectively. Red and blue edges mean increased or decreased co-expression between COPD and smokers, respectively. The full network with the dynamic layout and searchable gene names and functions in the HTML format is available at https://chuanxingli.github.io/pages/Sharing/FigS3.html and in Supplementary Figure S3. **(B)** Power-law degree distribution with the linear regression of degree (k) ∼ the probability of degree (P(k) in log10 scale) of linear regression R-squared = 0.943, *p*-value = 8.19*10–6. **(C)** Four major types of three-node motifs and their counts in the integrative network. Motifs 1 and 2 mean miRNAs significantly (FDR<=0.2) increased or decreased the regulation of mRNA transcription repression in COPD vs. smoker, respectively. Motifs 3 and 4 mean miRNAs significantly (FDR ≤0.2) increased or decreased the regulation of protein expression (potential protein translation inhibition) in COPD vs. smoker, respectively. The number under the motif is their count in the integrative regulated network. Created using igraph and Cytoscape. The full names of genes are provided in Supplementary Table S2.

The topological analysis includes calculation and identification of degrees (the number of connections of nodes), hubs (nodes with high degrees), betweenness centrality (the number of “shortest paths” going through nodes), bottlenecks (nodes with a high betweenness centrality), communities (densely connected subgraphs *via* random walks), and degree distribution using the R package igraph (Csardi and Nepusz, 2006; Barabasi et al., 2011). The three-node motifs (repeated triangle structure in Figure 2) were identified using an exhaustive method.

The power-law degree distribution (the probability distribution of these degrees over the whole network) was estimated through linear regression analysis with R-squared as the goodness-of-fit measure.

### 2.5 Functional enrichment analysis

Function enrichment analysis was performed for function terms from the Gene Ontology (biological process (BP), cellular component (CC), and molecular function (MF)) (Ashburner et al., 2000), KEGG (Kanehisa and Goto, 2000), Reactome (Jassal et al., 2020), WikiPathways (Martens et al., 2021) (exclude disease pathways), and MSigDB (Subramanian et al., 2005) databases by the over-representation analysis (ORA) method using the R package WebGestaltR (version 0.3.1) (Liao et al., 2019). ORA tested all terms from these databases with 5–500 gene annotations, and then enrichment FDR was estimated using the Benjamini–Hochberg method. The whole human genome was used as the reference genome (hg19). An enriched function graph was constructed, with function terms as nodes and overlaps (in the network) between terms as edges. The weights of edges were the numbers of shared genes in the integrative dysregulated network between each pair of function terms. The clusters of functions were identified as the network communities (densely connected subgraphs) *via* random walks using the R package igraph (Csardi and Nepusz, 2006; Barabasi et al., 2011).

### 2.6 miRNA disease gene knowledge databases

Information for all miRNAs in the dysregulated network was retrieved from 11 disease gene databases, namely, Gene2Function (Hu et al., 2017), KEGG (Kanehisa and Goto, 2000), MegGen (https://www.ncbi.nlm.nih.gov/medgen/9818), DISEASE (Pletscher-Frankild et al., 2015), VarfromPDB (https://cran.r-project.org/web/packages/VarfromPDB/), GAD (Becker et al., 2004), HMDD V3.0 (Huang et al., 2019), EDGAR (Babbi et al., 2017), DisGeNET v6 (Pinero et al., 2020), GeneCards (https://www.genecards.org/), and Disease Ontology (https://disease-ontology.org).

## 3 Results

### 3.1 Construction of the dysregulated miRNA–mRNA–protein network in COPD in females

An integrative dysregulated network was constructed, including 70 miRNAs, 66 mRNA, and 100 protein nodes linked by a total of 275 dysregulated edges (100 miRNA to mRNAs, 164 miRNAs to protein, and 11 mRNAs to protein dysregulation, see Figures 1D, 2A), as in the illustration of steps in Figure 1. The definition of “dysregulated” in this article is significantly differentially co-expressed patterns of two connected nodes between two statuses. Three miRNA dysregulated networks for COPD smokers *versus* smokers with normal lung functions (Gc), COPD smokers *versus* never-smoking healthy subjects (Gu), and smokers (with normal lung functions) *versus* (never-smoking) healthy subjects (Gs) with FDR ≤0.2 (see Figure 1C and the methods for dysregulated network construction) were constructed. Then, an integrative network among them was generated (subtraction of Gu and Gs and then intersection with Gc), which included the maximum use of the cohort and reduced the potential false-positive edges (the definitions of all these different networks are provided in Supplementary Table S2 and their degree distribution in Supplementary Figure S1). The integrative network was used as the miRNA–mRNA–protein dysregulated network in COPD in females and is referred to as the integrative dysregulated network in the following analysis (Figures 1D, 2A).

The integrative dysregulated network is selected as its maximal utilization of omics data and cohort information (all three groups of subjects), as well as the most analogy in the topological characteristics of the known biological networks with both mathematical and biological meanings. It is a small-scale graph with less than three hundred nodes and edges. Both hubs (with a high degree) and bottlenecks (with a high betweenness centrality) were important in the completeness and information transfer inside the network. It well matched the scale-free characteristics with the power-law degree distribution (the probability distribution of these degrees over the whole network) in most of the biological networks on a large scale (Figure 2B. R-squared = 0.943, *p*-value = 8.19*10–6). It also matched the modularity characteristics in most biological networks, which correspond to biological functions (Supplementary Figure S2: 18 densely connected communities; Supplementary Table S3: all topological features for each node). In summary, this is a well-connected, scale-free, and modularized small network.

### 3.2 Both abnormal regulations in miRNA-induced mRNA transcription and protein translation repression play roles in COPD

The dysregulated network is constructed by three-node motifs (repeated triangle structure) among miRNA, mRNA, and proteins in which the motifs themselves represent the two major mechanisms of miRNA regulation: mRNA transcription repression and protein translation inhibition (Bartel, 2004; Gebert and MacRae, 2019) (Figure 2C). Based on our hypothesis that the co-expression (defined by the Pearson correlation coefficient) indicates the regulation strengths, the dysregulation in these two regulation types can be represented by different three-node motifs in the network. In Figure 2C, four typical types of motifs are counted in the network. We found 127 dysregulations in protein translation inhibition and 82 dysregulations in mRNA transcription repression, which are the major three-node motif types in the network. These results indicated that both types of abnormal regulation play a role in the COPD mechanism. The number and percentage of each miRNA in each type of motif were presented in the character list to prioritize candidate risk miRNA (see the full table for all possible motifs in Supplementary Table S4).

### 3.3 The integrative network enriched in three clusters of functions: mitochondrial: ER, ER: Golgi: neutrophil, and miRNA: extra-pulmonary manifestations

**FIGURE 3:**
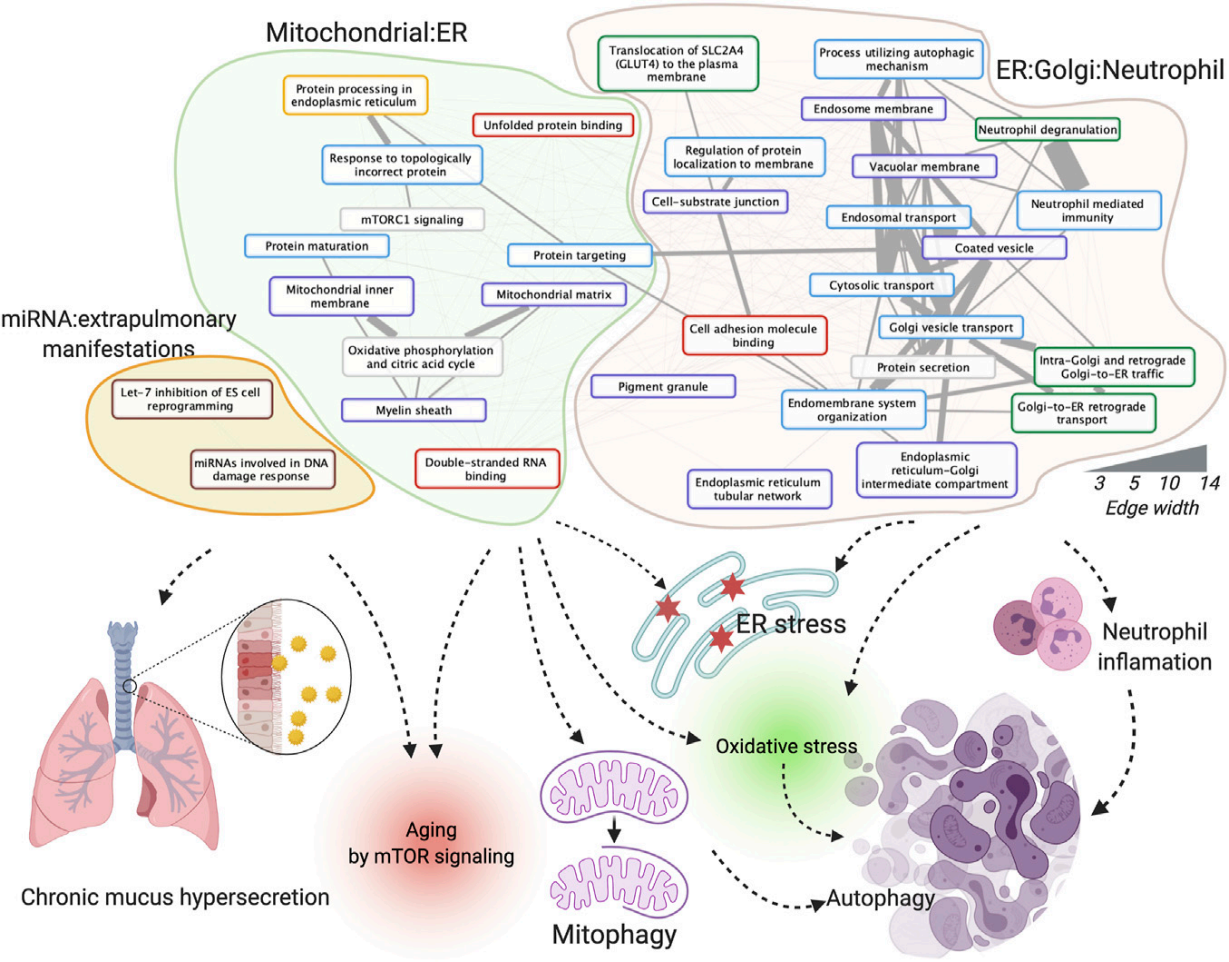
Enriched functions, function clusters, and their potential roles in COPD. The board colors of function terms are Hallmark50 (grey), Gene Ontology (GO) biological process (blue), GO cellular component (purple), GO molecular function (red), KEGG pathway (yellow), Reactome pathway (green), and WikiPathway (brown). The width of the edges corresponds to the number of co-annotated genes. Created using igraph, Cytoscape, and BioRender.com.

We investigated the enriched functions of this integrative dysregulated network in Gene Ontology (Ashburner et al., 2000), KEGG (Kanehisa and Goto, 2000), Reactome (Jassal et al., 2020), WikiPathway (Martens et al., 2021) (exclude disease pathways), and MSigDB (Subramanian et al., 2005) (see Materials and Methods). The correlation graph among the 36 enriched functions was further clustered into three functional clusters in COPD in females, 1) mitochondrial: endoplasmic reticulum (ER), 2) ER: Golgi: neutrophil, and 3) miRNA: extra-pulmonary manifestations (Figure 3). In brief, according to the literature analyses (Tasena et al., 2018, Dubinsky AN, et al. Cell Metab. 2014), the functions in the mitochondrial: ER cluster may contribute to COPD pathogenesis and progression through multiple ways and affects mTOR signaling, mitophagy, and autophagy. In addition to the aforementioned mechanism, the ER: Golgi: neutrophil cluster affects COPD by inflammatory changes from neutrophil products. The miRNA and extra-pulmonary manifestation cluster may affect COPD by chronic mucus hypersecretion and aging by mTOR signaling, for e.g., through let-7 inhibitions of cell reprogramming. A complete explanation of enriched functions’ roles in COPD with references and their enriched statistics and Entrez Gene ID are provided in Supplementary Table S5.

### 3.4 Prioritization of disease biomarkers by topological bottleneck indexes and further annotated by function and literature supports

**FIGURE 4:**
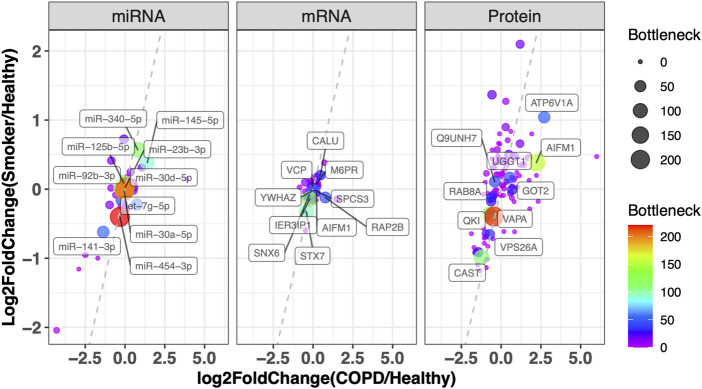
Prioritization of disease genes by topological character in the network and DEG in COPD. Plot of miRNA, mRNA, and protein with their log2 fold change in COPD vs. healthy (*x*-axis) and smoker vs. healthy (*y*-axis). The color and node size correspond to their bottleneck values in the Integrative Network. The symbols of the top 10 miRNAs, mRNAs, and proteins with the highest bottleneck values are labeled (the full node characters in the network and differentially expressed tests are provided in Supplementary Table S2). VCP: valosin-containing protein; YWHAZ: tyrosine 3-monooxygenase/tryptophan 5-monooxygenase activation protein, zeta; CALU: calumenin; SNX6: sorting nexin 6; M6PR: mannose-6-phosphate receptor, cation-dependent; IER3IP1: immediate early response 3-interacting protein 1; SPCS3: signal peptidase complex subunit 3; RAP2B: RAP2B, member of the RAS oncogene family; STX7: syntaxin 7; AIFM1: apoptosis-inducing factor mitochondria-associated 1; ATP6V1A: ATPase H+-transporting V1 subunit A; Q9UNH7: sorting nexin 6; UGGT1: UDP-glucose:glycoprotein glucosyltransferase 1; RAB8A: Ras-related protein Rab-8A; GOT2: glutamic oxaloacetic transaminase 2; QKI: quaking; VAPA: virulence-associated protein A; VPS26A: vacuolar protein sorting-associated protein 26a; CAST: calpastatin.

We further integrated the topological features, function and literature annotation, and prioritization of disease biomarkers. Information on all miRNAs in the dysregulated network was assembled from ten miRNA disease gene databases (see Materials and Methods). Twenty-two miRNAs have been reported by at least one database. As the coverage and annotation depths of the databases varied, these 22 miRNAs were validated manually (Supplementary Table S3). Further manual curation revealed 43 additional nodes being related to COPD. Based on our previous findings in prioritizing prostate cancer disease genes by topological features, the disease genes tend to link to other disease genes. In the integrative network, there are 50 genes connected to 24 literature-support nodes, which is more interesting for further investigation (see Supplementary Table S3). In Figure 4, genes are plotted by their differentially expressed ratios between COPD vs. smoker and COPD vs. healthy. The top 10 miRNAs, mRNAs, and proteins with the highest bottleneck values are labeled in Figure 4, which are our prioritized risk gene list.

### 3.5 Let-7-AIFM1-FKBP1A pathway in COPD pathology

Based on the definition of a status-specific dysregulated network, the network is only determined by the differences in the correlation of two nodes between two statuses but not by the nodes’ differential expression between two statuses. The differentially expressed gene (DEG, *p*-value ≤ 0.05) filter further extracts a subnetwork with 53 nodes and 45 edges with ten connected parts in Figure 1E. The poor connection of the DEG subnetwork further emphasizes the importance of the construction of the integrative network without including DEG nodes only.

**FIGURE 5:**
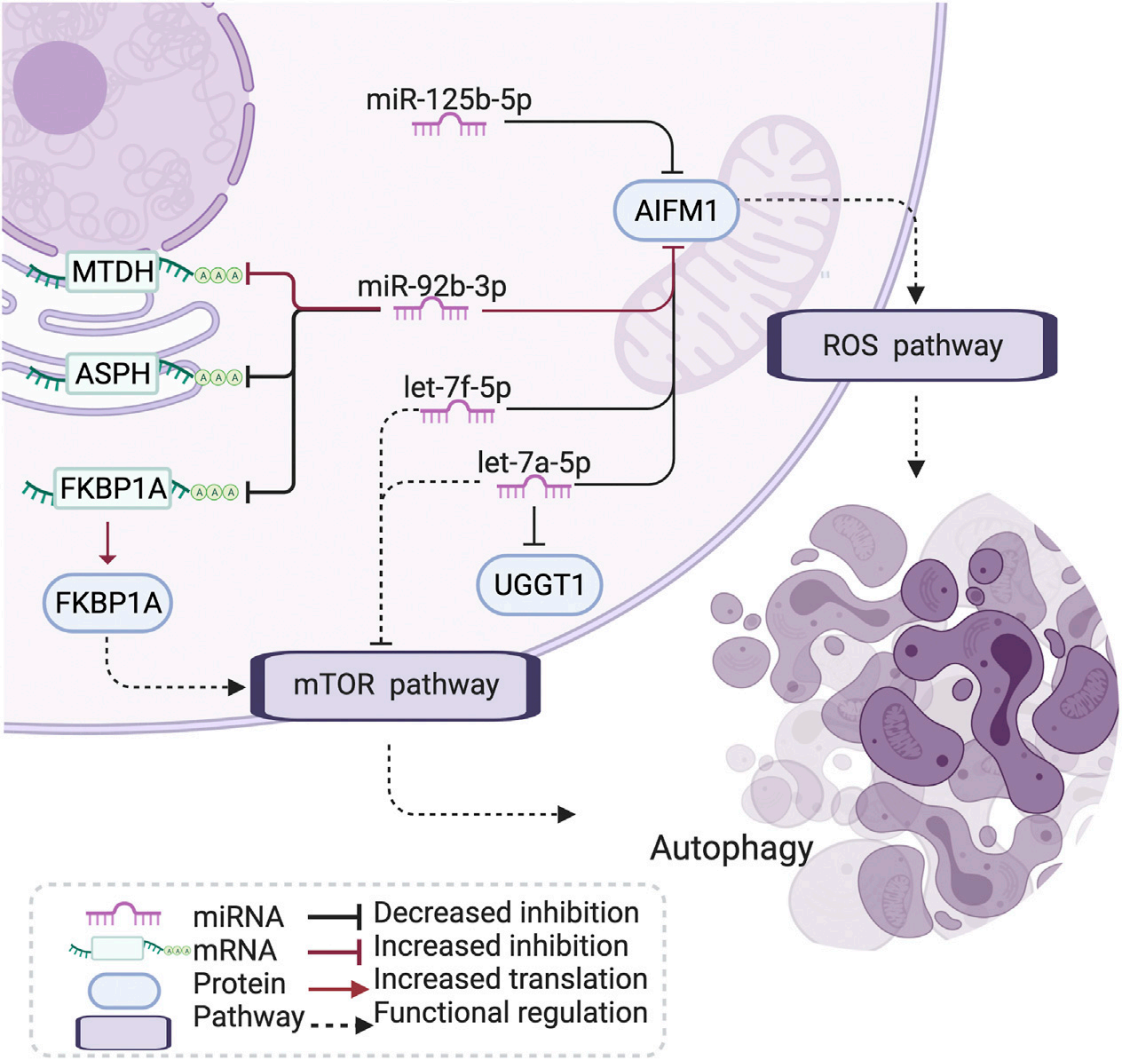
Let-7-AIFM1-FKBP1A pathway and its potential effect in COPD. The let-7 family has an increased correlation with AIFM1 protein expression in COPD, which may induce a stronger inhibition than through the ROS and mTOR pathway to influence autophagy and cell differentiation in COPD. AIFM1: apoptosis-inducing factor mitochondria-associated 1, FKBP1: FKBP prolyl isomerase 1A, MTDH: metadherin, ASPH: aspartate β-hydroxylase, UGGT1: UDP-glucose:glycoprotein glucosyltransferase 1, ROS: reactive oxygen species, mTOR: mammalian target of rapamycin, COPD: chronic obstructive pulmonary disease. Created using BioRender.com.

Notably, literature analyses showed that the miRNA let-7–apoptosis-inducing factor mitochondria-associated 1 (AIFM1)–FKBP prolyl isomerase 1A (FKBP1A) pathway shows connections with the regulation of apoptosis and autophagy in disease (Araki et al., 2009; Yoshida et al., 2010; Dubinsky et al., 2014; Holze et al., 2018; Houssaini et al., 2018; Tasena et al., 2018) (Figure 5). In brief, the let-7 family could downregulate the expression of amino acid-sensing pathway genes to repress mTORC1 and is involved in autophagy, and FKBP1A is related to the mTOR pathway to regulate memory T-cell differentiation. AIFM1 is associated with the reactive oxygen species (ROS) pathway (Figure 5)

## 4 Discussion

Here, we investigated the dysregulation of the miRNA–mRNA–protein axis in COPD based on the integration of triple-omics expression and regulatory networks. Our focus was placed on the dysregulation (differential co-expression) in women with mild-to-moderate, smoking-induced COPD. Based on the constructed differential network, biomarkers and their topological characteristics were prioritized and enriched into several at-risk biological functions and cellular components. Network motif analysis indicated that both miRNA-induced alterations in mRNA and protein abundance may play roles in COPD. Finally, downstream analysis identified that the let-7-AIFM1-FKBP1A pathway, through ROS and the mTOR pathway, may influence autophagy and cell differentiation in COPD.

Several factors may influence the outcome of the dysregulated network, including sample size, study group homogeneity, and the coverage and accuracy of the reference regulatory network. Whereas a larger cohort would facilitate more robust molecular insights, the availability of cohorts that offer multi-omics data collected from the lung and the site of inflammation is scarce. The homogenous nature of our Karolinska COSMIC cohort, with the application of strict inclusion and exclusion criteria to generate a cohort of early-stage COPD patients naive of prior treatment and with no comorbidities, as well as the focus on the female sex only, aids to improve the statistical power despite the small sample sizes. We have previously shown that multi-omics integration can improve the statistical power in small group sizes (Li et al., 2018). In this study, we attempted to investigate COPD in a network-based, edge/interaction-focused, and multi-omics integration fashion as a complement to more traditional single-omics, single-marker, gene-focused research.

The let-7 family plays an important role in the regulation of chronic mucus hypersecretion, which has been associated with a worse prognosis and quality of life in COPD (Tasena et al., 2018). The let-7 family has been shown to downregulate the expression of amino acid-sensing pathway genes to repress mTORC1 (Dubinsky et al., 2014). mTOR signaling has been associated with cigarette smoke (CS)-induced COPD/emphysema through its crucial role in regulating autophagy (Yoshida et al., 2010; Kim and Guan, 2015) and inducing cell senescence in COPD (Houssaini et al., 2018). Studies have shown that autoreactive T cells are present in ex-smokers with emphysema, and the degree of their activation is closely related to impaired lung function (Xu et al., 2012). AIFM1 is a proapoptotic factor, binding with the partner of the phosphatase PGAM5. AIFM1 and PGAM5 are associated with ROS-induced cell-death signaling (Holze et al., 2018). Apoptosis of lung structural cells is an important upstream event in the pathogenesis of COPD (Demedts et al., 2006), involving the destruction of lung tissue and the development of emphysema (Song et al., 2021). In addition, epigenetic and other molecular biological mechanisms presented the role in apoptosis of pulmonary vascular endothelial cells (Song et al., 2021). Also, the excessive generation of mitochondrial ROS has been indicated to promote chronic inflammation of the airways (Jiang et al., 2017). miR-92b-3p, one of the identified drivers in the differential network, has been reported to take part in COPD and several other diseases by regulating proliferation, apoptosis, and differentiation (Hao et al., 2018). In addition, hypoxia-induced miR-92b-3p is indicated as a potent regulator of the mTOR signaling pathway (Lee et al., 2019). FKBP1A plays a role in antigen-specific CD8 T cells and is related to the mTOR pathway to regulate memory T-cell differentiation (Araki et al., 2009). The subnetwork of let-7-AIFM1-FKBP1A thus explains a potential mechanism of oxidative stress, ROS, and apoptosis in COPD pathology.

Here, we presented a systematic multi-omics and regulatory network integration study to construct a miRNA–mRNA–protein dysregulation network for COPD based on the female subjects in our Karolinska COSMIC cohort. Each miRNA is predicted to target multiple mRNAs, and conversely, each mRNA can be targeted by many different miRNAs (Di Leva et al., 2014). Although miRNAs under certain circumstances can activate protein translation (O'Brien et al., 2018), this study focused on the more commonly induced suppression of protein translation. As such, only negative regulations between miRNA and mRNA were selected for further analysis. The utilized modelling approach revealed significantly differentially co-expressed patterns of miRNA-to-mRNA and miRNA-to-protein in COPD. Furthermore, downstream network topology, literature annotation, and functional enrichment analysis prioritized both known and novel disease-related biomarkers and pathways. Abnormal regulations in miRNA-induced mRNA transcription and protein translation repression were found to play roles in COPD. Specifically, the let-7-AIFM1-FKBP1A pathway is highlighted in COPD pathology. This study presents a means of molecular mechanistic modeling of COPD at the multi-omics level. The improved statistical power achieved by an integration of molecular information from multiple levels harbors the potential to facilitate the identification of putative molecular diagnostic, prognostic, or treatment targets also in relatively small cohorts, particularly if the cohorts are well-designed to isolate specific disease sub-phenotypes of patients.

## Data Availability

The original contributions presented in the study are included in the article/Supplementary Material; the data presented in the study are deposited in the Swedish National Data Service repository, accession number 2022-172 (https://snd.gu.se/en/catalogue/study/preview/764893f7-d9e8-4f95-b1f7-2f163adfec9f); further inquiries can be directed to the corresponding authors.
