# Supplementary material for: miRNA–mRNA–protein dysregulated network in COPD in women
Source: Front Genet. 2022 Nov 17;13:1010048. doi: 10.3389/fgene.2022.1010048 (PMC9712209; doi:10.3389/fgene.2022.1010048)
Supplement: Supplementary file 1 [file Presentation1.zip › supplementary material/Supplementary_Material.docx]

Supplementary Material

# Supplementary Figures and Tables

## Supplementary Figures

**Figure S1**. The power-law distribution of 5 networks in the rows (detailed definition in Supplementary Table S1) with 3 FDR thresholds in the columns. FDR.05, FDR.1, and FDR.2 represent the three FDR thresholds 0.05, 0.1, and 0.2 respectively. The X-axis represents the node degree, and the Y-axis is their percentage in logarithmic 10 scale. The blue lines are the linear regressions of *k* ~ P(k) in logarithmic scale with p-value (p) of Pearson correlation test and R-squared (R2) of Pearson correlation coefficients. The power-law distribution is a feature of scale-free network, which is also found in most biological networks. In this study, both Gi and Gt with FDR <= 0.2 are strong power-law distribution, and considering the maximum use of cohort and reducing the potential false-positive edges, Gt with FDR <= 0.2 is selected as "Integrative Dysregulated Network" in further analysis.

**Figure S2**. 18 network communities in the "Integrative Dysregulated Network" (Gt with FDR <= 0.2) by the spin-glass model and simulated annealing method in the R package igraph. The detailed cluster number for each gene can be found in Supplementary Table S2.

**Figure S3**. The complete Integrative Dysregulated Network with dynamic layout and searchable gene names and functions in HTML format. The integrative dysregulated network is a directed network from miRNA (red nodes) to mRNA (blue nodes), mRNA to protein (yellow nodes) or from miRNA to protein (see legend at right). Nodes with red, blue and yellow borders represent miRNA, mRNA and protein respectively. Nodes in rectangles are annotated COPD-related genes from the disease database search (details of descriptions in Supplementary Table S2). Nodes’ sizes correspond to their Bottleneck values in the network. Red and blue edges indicated increased and decreased coexpression between COPD and Smokers, respectively. The network can be easily enlarged or reduced. After clicking on a node, the clicked node and its connected nodes are highlighted, and the left part of the network is in greyed out. There are two ways to search this network: “Select by id” for gene names, and “Select by group” for function terms. The searchable function terms correspond to the enriched functions in Table S5. Created with the igraph and visNetwork package in R (https://datastorm-open.github.io/visNetwork/, see FigS3.html).

## Supplementary Tables

**Table S1.** Three status dysregulated- and two integrative-networks’ symbols, meanings and calculation methods.

| Network  Symbols | FDR <= | | | Meaning | Calculation |
| --- | --- | --- | --- | --- | --- |
|  | **0.05** | **0.1** | **0.2** |  |  |
| $\boldsymbol{G}_{\boldsymbol{c}}$ | $G_{c}^{.05}$ | $G_{c}^{.1}$ | $G_{c}^{.2}$ | Dysregulated network in COPD given smoking | COPD vs. Smoker |
| $\boldsymbol{G}_{\boldsymbol{u}}$ | $G_{u}^{.05}$ | $G_{u}^{.1}$ | $G_{u}^{.2}$ | Dysregulated network in COPD or smoking compared to healthy | COPD vs. Healthy |
| $\boldsymbol{G}_{\boldsymbol{s}}$ | $G_{s}^{.05}$ | $G_{s}^{.1}$ | $G_{s}^{.2}$ | Dysregulated network in smoker verse healthy | Smoker vs. Healthy |
| $\boldsymbol{G}_{\boldsymbol{i}}$ | $G_{i}^{.05}$ | $G_{i}^{.1}$ | $G_{i}^{.2}$ | Shared network by $\boldsymbol{G}_{\boldsymbol{c}}$ and $\boldsymbol{G}_{\boldsymbol{u}}$ (increase the possibility by adding more samples) | $G_{c}\cap G_{u}$ |
| $\boldsymbol{G}_{\boldsymbol{t}}$ | $G_{t}^{.05}$ | $G_{t}^{.1}$ | $G_{t}^{.2}$ | Shared network between $\boldsymbol{G}_{\boldsymbol{c}}$ and the possible COPD only influenced dysregulated network by the relative complement of $\boldsymbol{G}_{\boldsymbol{s}}$ in $\boldsymbol{G}_{\boldsymbol{u}}$ | $G_{c}\cap(G_{u}-G_{s})$ |

**Table S2**. Node attributes in the Integrative Dysregulated Network, including Fold Change in COPD, Smokers and Healthy and Degree, Bottleneck, and whether supported by database annotations to be COPD-related genes (see Description sheet in TableS2.xlsx).

**Table S3**. All appeared 3-node motifs in the integrative network. -, ↑ and ↓represent insignificant changes, significantly increased and decreased coexpression between COPD and smokers, respectively (FDR <= 0.2). The “Counts” column is the total number of motifs occurring in the integrative dysregulated network (Corresponding to Figure 2c).

| Motifs | miR->mRNA | mRNA->Protein | miR->Protein | Counts |
| --- | --- | --- | --- | --- |
| 1 | - | - | ↓ | 89 |
| 2 | - | ↑ | - | 71 |
| 3 | - | - | ↑ | 57 |
| 4 | - | ↓ | - | 56 |
| 5 | ↓ | - | - | 50 |
| 6 | ↑ | - | - | 32 |
| 7 | ↑ | ↑ | - | 5 |
| 8 | ↓ | ↑ | - | 5 |
| 9 | - | ↑ | ↑ | 5 |
| 10 | - | ↓ | ↓ | 4 |
| 11 | ↑ | - | ↑ | 3 |
| 12 | - | ↑ | ↓ | 3 |
| 13 | ↓ | - | ↓ | 2 |
| 14 | ↓ | ↓ | - | 2 |
| 15 | ↑ | ↓ | - | 1 |
| 16 | - | ↓ | ↑ | 1 |

**Table S4.** Full explanation of enriched functions’ roles in COPD with references.

| **Clusters** | **Function Terms** | **Function Description** | **References** | **Role in COPD** | **References** |
| --- | --- | --- | --- | --- | --- |
| 1 | oxidative phosphorylation and citric acid cycle | Long-term cigarette smoke exposure increases the expression of fission/fusion markers, oxidative phosphorylation proteins and markers of oxidative stress. | Hoffmann, R. F, et al. Respir Res 2013 | - | - |
| 1 | mTORC1 signaling | 1. Activation of the mTORC1/PGC-1 axis promotes mitochondrial biogenesis and induces cellular senescence in the lung epithelium. | Summer R, et al. Am J Physiol Lung Cell Mol Physiol. 2019. | 1. mitochondrial dysfunction has emerged as an important causative factor for cellular senescence. cellular senescence contributes to the development of COPD. | 1 Summer R, et al. Am J Physiol Lung Cell Mol Physiol. 2019. |
|  |  | 2. Blocking the mTORC1/PGC-1α/β axis or reducing ROS-induced molecular damage could be effective in the treatment of senescence-associated lung diseases |  | 2. Autophagy is enhanced in the locomotor muscles of patients with COPD. Enhanced autophagy is associated with inhibition of the mTORC1 pathways, and the development of oxidative stress. | 2. Guo Y, et al, Am J Respir Crit Care Med. 2013 |
| 1 | mitochondrial inner membrane | 1. The IM is equipped with a series of highly conserved, proteolytic complexes dedicated to the maintenance of normal protein homeostasis within this mitochondrial subcompartment. | 1. Levytskyy RM, et al. Biochemistry. 2017 Sep | 1. PHB1 downregulation in COPD patients and that PHB1 expression levels are associated with the degree of airway obstruction. | Soulitzis N, et al. Respir Med. 2012 |
|  |  | 2. Prohibitins (PHB1 and PHB2) are versatile proteins located at the inner mitochondrial membrane, maintaining normal mitochondrial function and morphology. | 2. Soulitzis N, et al. Respir Med. 2012 | 2. The significant downregulation of PHB1 in COPD and non-COPD smokers in comparison to non-smokers possibly reflects a distorted mitochondrial function due to decreased mitochondrial stability, especially in the mitochondria of COPD patients. |  |
| 1 | mitochondrial matrix | The mitochondrial matrix integrates the folding and assembly of proteins derived from the nuclear and mitochondrial genomes. | Munch, C, et al. Nature. 2016 | - | - |
| 1 | response to topologically incorrect protein | 1. The topology of the sites of superoxide production in mammalian mitochondria is important because it determines whether or not a site will produce superoxide in the mitochondrial matrix and be able to damage mitochondrial DNA. | 1. Brand, M. D et al, Exp Gerontol 2010, | - | - |
|  |  | 2. Topology of superoxide production from different sites in the mitochondrial electron transport chain | 2. St-Pierre, J et al. J Biol Chem 2002 | - | - |
| 1 | unfolded protein binding | ER stress leads to activation of the unfolded protein response (UPR) signaling cascade and induction of an apoptotic cell death, autophagy. | Sakabe, I., et al. Breast Cancer Res Treat 2015. | Damaged proteins are present in the COPD lung, that elimination of these damaged proteins is impaired, and that the UPR may play a role in the pathogenesis of COPD | Guo, Y, et al . Am J Respir Crit Care Med. 2013 |
| 1 | protein targeting | Regulate mitochondrial fission are dynamin-related protein 1 (DRP1) and fission protein 1 homolog (FIS1) | Liesa, M. et al. Physiol Rev 2009 | - | - |
| 1 | protein maturation | UPR can be triggered by a broad range of stressful conditions which disrupt successful maturation of proteins in the endoplasmic reticulum (ER) by interfering with proper folding, assembly, and posttranslational modification. | Zhao, H. et al. Methods Enzymol, 2011 | - | - |
| 1 | double-stranded RNA binding | RNA-binding proteins provide a uniquely complex regulatory code that orchestrates mitochondrial function during physiological and pathological conditions. | Schatton, D. et al. Crit Rev Biochem Mol Biol 2018 | Oxidative stress enhances toll-like receptor 3 response to double-stranded RNA in airway epithelial cells | Koarai, A. et al. Am J Respir Cell Mol Biol. 2010 |
| 1 | Protein processing in endoplasmic reticulum | Protein processing in endoplasmic reticulum | - | 1. The inhibition of autophagy could attenuate the endoplasmic reticulum stress (ERS) -induced apoptosis of alveolar epithelial cells (AECs)in rats with COPD. | 1. Tang, Y. et al. Biosci Rep 2017 |
|  |  |  |  | 2. Accumulation of nonfunctional and potentially cytotoxic, misfolded proteins in COPD is believed to contribute to lung cell apoptosis, inflammation, and autophagy. | 2. Kelsen, S. G. et al. Ann Am Thorac Soc 2016 |
| 1 | myelin sheath | ER stress and the UPR play a role in a number of disorders of myelin and myelinating glia | Clayton, B. L. L. et al. Brain Res 2016 | - | - |
| 2 | protein secretion | Conventional protein secretion (CPS) is the trafficking route that secretory proteins undertake when are transported from the endoplasmic reticulum (ER) to the Golgi apparatus (GA), and subsequently to the plasma membrane (PM) via secretory vesicles or secretory granules. | Viotti, C. et al.Methods Mol Biol 2016 | - | - |
| 2 | regulation of protein localization to membrane | Proteins are translated in the ER, where they can then be modified and packaged into vesicles bound for the Golgi complex. Through the Golgi, transported proteins can be further modified before sorting to their final localization at the lysosome or plasma membrane where the proteins function. | Weidner, J. et al. Physiol Rep 2018 | - | - |
| 2 | process utilizing autophagic mechanism | persistent ER stress often results in stimulation of autophagic activities | Luchetti, F. et al. Redox Biol. 2017 | 1. Excessive autophagy and mitophagy induced by cigarette smoke results in bronchial cell apoptosis and necroptosis, respectively, thus providing a possible mechanism of emphysema. | 1.Chen, Z. H. et al. Proc Natl Acad Sci USA 2010 |
|  |  |  |  | 2. Insufficient autophagy promotes bronchial epithelial cell senescence in chronic obstructive pulmonary diseas | 2. Fujii, S. et al. Oncoimmunology 2012 |
| 2 | endoplasmic reticulum tubular network | The endoplasmic reticulum comprises morphologically distinct domains, including a dynamic network of interconnected membrane tubules. | Powers, R. E. et al. Nature 2017 | Endoplasmic reticulum is disorganized in COPD patients | 1. Weidner, J. et al. Physiol Rep. 2018 |
| 2 | endoplasmic reticulum-Golgi intermediate compartment | The ER-Golgi intermediate compartment is a key membrane source for the LC3 lipidation step of autophagosome biogenesis. | Ge, L. et al. Elife 2013 | Golgi is altered in COPD patients | 1. Weidner, J. et al. Physiol Rep. 2018 |
| 2 | Golgi vesicle transport | The Golgi apparatus occupies a central position within the secretory pathway where it is a hub for vesicle trafficking. | 1. Witkos, T. M. et al. Current Opinion in Cell Biology 2017 | - | - |
| 2 | Intra-Golgi and retrograde Golgi-to-ER traffic | Secretory proteins are transported from the ER, through the Golgi and into the trans-Golgi network (TGN) where they are sorted to either the endosomal/lysosomal system or the plasma membrane for secretion | 1. Griffiths, G et al. Science 1986 2. Cottam, N. P. et al. Protoplasma. 2012 | - | - |
| 2 | Golgi-to-ER retrograde transport |  |  | - | - |
| 2 | endomembrane system organization | endomembrane pathway includes the endoplasmic reticulum (ER), Golgi, and lysosomes. | Weidner, J. et al. Physiol Rep 2018 | COPD patients have phenotypic changes in the lung fibroblasts endomembrane pathway, and respond differently to stress. | Weidner, J. et al. Physiol Rep 2018 |
| 2 | endosomal transport | The early endosome receives all manner of incoming material from the plasma membrane, as well as from the Golgi.The late endosome provides a outgoing traffic to the lysosomes, the Golgi complex or the plasma membrane. | Scott, C. C. et al. Seminars in Cell & Developmental Biology 2014 | - | - |
| 2 | cytosolic transport | Protein sorting requires assembly of the cytosolic sorting machinery onto the trans-Golgi network membrane and capture of cargo proteins. | Guo Y. et al. Annu Rev Cell Dev Biol. 2014 | - | - |
| 2 | cell-substrate junction | Epithelial barrier function is maintained by intercellular contact formation in so-called adherens junctions (AJs) and tight junctions (TJs). | Heijink IH. et al. Eur Respir J. 2010 | The airway epithelium plays a central role in the pathogenesis of various pulmonary diseases, including asthma, COPD and cystic fibrosis. | Heijink IH. et al. Eur Respir J. 2010 |
| 2 | vacuolar membrane | Membrane proteins in the endoplasmic reticulum (ER) or in the Golgi apparatus would automatically proceed downstream to the plasma or vacuole membranes | Gao C. et al. Trends Plant Sci. 2014 | - | - |
| 2 | endosome membrane |  |  | - | - |
| 2 | Translocation of SLC2A4 (GLUT4) to the plasma membrane | GLUT4 is translated within the endoplasmic reticulum, then transported to the trans Golgi network, then inserted into budding GLUT4-storage vesicles (GSV) vesicles. Insulin stimulation releases GSVs from storage, and they are transported to the plasma membrane. | 1.Sadler JB. et al. Int J Mol Sci. 2013 | GLUT4 was reduced in COPD. | Green HJ. et al. J Appl Physiol . 2008 |
| 2 | neutrophil mediated immunity | Neutrophil | - | Neutrophil-associated COPD with activation of the inflammasome | Brightling C. et al. Eur Respir J. 2019 |
| 2 | Neutrophil degranulation |  |  | Neutrophil products are thought to be key mediators of inflammatory changes in the airways of COPD patients, including impaired migratory accuracy and increased degranulation and reactive oxygen species release. | 1. Butler A. et al. COPD. 2018 |
| 2 | pigment granule | - |  | Hemosiderin in sputum macrophages may predict infective exacerbations of chronic obstructive pulmonary disease | 1. Mohan S. et al. BMC Pulm Med. 2017 |
| 2 | cell adhesion molecule binding | Triglyceride-rich lipoprotein modulates endothelial vascular cell adhesion molecule (VCAM)-1 expression via differential regulation of endoplasmic reticulum stress. | Wang YI. et al. PLoS One. 2013 | Adherens junction proteins are altered following cigarette smoke exposure and in COPD patients | Nishida, K. et al. Am J Physiol Lung Cell Mol Physiol. 2017 |
| 3 | miRNAs involved in DNA damage response | - | - | 1. Certain combinations of mitochondrial DNA (mtDNA) mutations (haplogroups) increase, whereas others decrease the risk of COPD. | 1. Mercado, N. et al. Thorax. 2015 |
|  |  |  |  | 2. Dysregulation of microRNAs (miRNAs) is involved in the pathogenesis of COPD, which is mainly resulted from cigarette smoke exposure (CSE) | 2. Dang, X. M. et al. Chem Biol Interact. 2019 |
| 3 | Metastatic brain tumor | - |  | Brain damage and cardiovascular disease are extra-pulmonary manifestations of COPD. | Spilling, C. A. et al. Int J Chron Obstruct Pulmon Dis. 2019 |
| 3 | let-7 inhibition of ES cell reprogramming | - |  | Let-7 family as regulators of chronic mucus hypersecretion in COPD | Tasena, H. et al. Eur Respir J. 2018 |
| 3 | Role of Osx and miRNAs in tooth development | Osterix (Osx) and miRNAs are needed for odontoblast differentiation and tooth development. | Wang, C. et al. Medical Science Monitor 2016 | Periodontitis Is Associated with Chronic Obstructive Pulmonary Disease | Takeuchi, K. et al.Journal of Dental Research 2019 |

**Table S5**. Significantly enriched function terms (FDR <= 0.05) of the Integrative Dysregulated Network, including annotated Entrez GeneID and link of each term(see independent EXCEL file TableS5.xlsx).

**References:**

Brand MD. The sites and topology of mitochondrial superoxide production. Exp Gerontol. 2010;45(7-8):466-472.

Brightling C, Greening N. Airway inflammation in COPD: progress to precision medicine. Eur Respir J. 2019;54(2).

Butler A, Walton GM, Sapey E. Neutrophilic Inflammation in the Pathogenesis of Chronic Obstructive Pulmonary Disease. Copd-Journal of Chronic Obstructive Pulmonary Disease. 2018;15(4):392-404.

Chen ZH, Lam HC, Jin Y, et al. Autophagy protein microtubule-associated protein 1 light chain-3B (LC3B) activates extrinsic apoptosis during cigarette smoke-induced emphysema. Proc Natl Acad Sci U S A. 2010;107(44):18880-18885.

Clayton BLL, Popko B. Endoplasmic reticulum stress and the unfolded protein response in disorders of myelinating glia. Brain Res. 2016;1648(Pt B):594-602.

Cottam NP, Ungar D. Retrograde vesicle transport in the Golgi. Protoplasma. 2012;249(4):943-955.

Dang XM, Yang L, Guo JX, et al. miR-145-5p is associated with smoke-related chronic obstructive pulmonary disease via targeting KLF5. Chem-Biol Interact. 2019;300:82-90.

Dubinsky AN, Dastidar SG, Hsu CL, et al. Let-7 Coordinately Suppresses Components of the Amino Acid Sensing Pathway to Repress mTORC1 and Induce Autophagy. Cell Metabolism. 2014;20(4):626-638.

Fujii S, Hara H, Araya J, et al. Insufficient autophagy promotes bronchial epithelial cell senescence in chronic obstructive pulmonary disease. Oncoimmunology. 2012;1(5):630-641.

Gao CJ, Cai Y, Wang YJ, et al. Retention mechanisms for ER and Golgi membrane proteins. Trends Plant Sci. 2014;19(8):508-515.

Ge L, Melville D, Zhang M, Schekman R. The ER-Golgi intermediate compartment is a key membrane source for the LC3 lipidation step of autophagosome biogenesis. Elife. 2013;2.

Green HJ, Bombardier E, Burnett M, et al. Organization of metabolic pathways in vastus lateralis of patients with chronic obstructive pulmonary disease. Am J Physiol Regul Integr Comp Physiol. 2008;295(3):R935-941.

Green HJ, Burnett ME, D'Arsigny CL, O'Donnell DE, Ouyang J, Webb KA. Altered metabolic and transporter characteristics of vastus lateralis in chronic obstructive pulmonary disease. J Appl Physiol. 2008;105(3):879-886.

Griffiths G, Simons K. The Trans Golgi Network - Sorting at the Exit Site of the Golgi-Complex. Science. 1986;234(4775):438-443.

Guo Y, Gosker HR, Schols AM, et al. Autophagy in locomotor muscles of patients with chronic obstructive pulmonary disease. Am J Respir Crit Care Med. 2013;188(11):1313-1320.

Guo YS, Sirkis DW, Schekman R. Protein Sorting at the trans-Golgi Network. Annu Rev Cell Dev Bi. 2014;30:169-206.

Heijink IH, Brandenburg SM, Noordhoek JA, Postma DS, Slebos DJ, van Oosterhout AJM. Characterisation of cell adhesion in airway epithelial cell types using electric cell-substrate impedance sensing. Eur Respir J. 2010;35(4):894-903.

Hoffmann RF, Zarrintan S, Brandenburg SM, et al. Prolonged cigarette smoke exposure alters mitochondrial structure and function in airway epithelial cells. Respir Res. 2013;14:97.

Jiang Y, Wang XQ, Hu DD. Mitochondrial alterations during oxidative stress in chronic obstructive pulmonary disease. Int J Chronic Obstr. 2017;12:1153-1162.

Kelsen SG. The Unfolded Protein Response in Chronic Obstructive Pulmonary Disease. Ann Am Thorac Soc. 2016;13 Suppl 2:S138-145.

Koarai A, Sugiura H, Yanagisawa S, et al. Oxidative stress enhances toll-like receptor 3 response to double-stranded RNA in airway epithelial cells. Am J Respir Cell Mol Biol. 2010;42(6):651-660.

Levytskyy RM, Bohovych I, Khalimonchuk O. Metalloproteases of the Inner Mitochondrial Membrane. Biochemistry. 2017;56(36):4737-4746.

Liesa M, Palacin M, Zorzano A. Mitochondrial dynamics in mammalian health and disease. Physiol Rev. 2009;89(3):799-845.

Luchetti F, Crinelli R, Cesarini E, et al. Endothelial cells, endoplasmic reticulum stress and oxysterols. Redox Biol. 2017;13:581-587.

Mercado N, Ito K, Barnes PJ. Accelerated ageing of the lung in COPD: new concepts. Thorax. 2015;70(5):482-489.

Mohan S, Ho T, Kjarsgaard M, et al. Hemosiderin in sputum macrophages may predict infective exacerbations of chronic obstructive pulmonary disease: a retrospective observational study. Bmc Pulmonary Medicine. 2017;17.

Munch C, Harper JW. Mitochondrial unfolded protein response controls matrix pre-RNA processing and translation. Nature. 2016;534(7609):710-713.

Nishida K, Brune KA, Putcha N, et al. Cigarette smoke disrupts monolayer integrity by altering epithelial cell-cell adhesion and cortical tension. Am J Physiol-Lung C. 2017;313(3):L581-L591.

Powers RE, Wang S, Liu TY, Rapoport TA. Reconstitution of the tubular endoplasmic reticulum network with purified components. Nature. 2017;543(7644):257-260.

Sadler JBA, Bryant NJ, Gould GW, Welburn CR. Posttranslational Modifications of GLUT4 Affect Its Subcellular Localization and Translocation. Int J Mol Sci. 2013;14(5):9963-9978.

Sakabe I, Hu R, Jin L, Clarke R, Kasid UN. TMEM33: a new stress-inducible endoplasmic reticulum transmembrane protein and modulator of the unfolded protein response signaling. Breast Cancer Res Treat. 2015;153(2):285-297.

Schatton D, Rugarli EI. A concert of RNA-binding proteins coordinates mitochondrial function. Crit Rev Biochem Mol Biol. 2018;53(6):652-666.

Scott CC, Vacca F, Gruenberg J. Endosome maturation, transport and functions. Semin Cell Dev Biol. 2014;31:2-10.

Soulitzis N, Neofytou E, Psarrou M, et al. Downregulation of lung mitochondrial prohibitin in COPD. Respir Med. 2012;106(7):954-961.

Spilling CA, Bajaj MPK, Burrage DR, et al. Contributions of cardiovascular risk and smoking to chronic obstructive pulmonary disease (COPD)-related changes in brain structure and function. Int J Chronic Obstr. 2019;14:1855-1866.

St-Pierre J, Buckingham JA, Roebuck SJ, Brand MD. Topology of superoxide production from different sites in the mitochondrial electron transport chain. J Biol Chem. 2002;277(47):44784-44790.

Summer R, Shaghaghi H, Schriner D, et al. Activation of the mTORC1/PGC-1 axis promotes mitochondrial biogenesis and induces cellular senescence in the lung epithelium. Am J Physiol Lung Cell Mol Physiol. 2019;316(6):L1049-L1060.

Takeuchi K, Matsumoto K, Furuta M, et al. Periodontitis Is Associated with Chronic Obstructive Pulmonary Disease. J Dent Res. 2019;98(5):534-540.

Tang Y, Cai QH, Wang YJ, et al. Protective effect of autophagy on endoplasmic reticulum stress induced apoptosis of alveolar epithelial cells in rat models of COPD. Biosci Rep. 2017;37(6).

Tasena H, Faiz A, Timens W, et al. microRNA-mRNA regulatory networks underlying chronic mucus hypersecretion in COPD. Eur Respir J. 2018;52(3).

Viotti C. ER to Golgi-Dependent Protein Secretion: The Conventional Pathway. Methods Mol Biol. 2016;1459:3-29.

Wang C, Liao HQ, Cao ZG. Role of Osterix and MicroRNAs in Bone Formation and Tooth Development. Med Sci Monitor. 2016;22.

Wang YI, Bettaieb A, Sun CX, et al. Triglyceride-Rich Lipoprotein Modulates Endothelial Vascular Cell Adhesion Molecule (VCAM)-1 Expression via Differential Regulation of Endoplasmic Reticulum Stress. Plos One. 2013;8(10).

Weidner J, Jarenback L, Aberg I, et al. Endoplasmic reticulum, Golgi, and lysosomes are disorganized in lung fibroblasts from chronic obstructive pulmonary disease patients. Physiol Rep. 2018;6(5).

Witkos TM, Lowe M. Recognition and tethering of transport vesicles at the Golgi apparatus. Curr Opin Cell Biol. 2017;47:16-23.

Zhao H, Yang J, Shan L, Jorgensen ED. Measuring the impact of cigarette smoke on the UPR. Methods Enzymol. 2011;489:147-164.
